# Renin-angiotensin system acting on reactive oxygen species in paraventricular nucleus induces sympathetic activation *via* AT1R/PKCγ/Rac1 pathway in salt-induced hypertension

**DOI:** 10.1038/srep43107

**Published:** 2017-03-24

**Authors:** Qing Su, Chan-Juan Huo, Hong-Bao Li, Kai-Li Liu, Xiang Li, Qing Yang, Xin-Ai Song, Wen-Sheng Chen, Wei Cui, Guo-Qing Zhu, Xiao-Lian Shi, Jin-Jun Liu, Yu-Ming Kang

**Affiliations:** 1 Department of Physiology and Pathophysiology, Xi’an Jiaotong University School of Basic Medical Sciences, Key Laboratory of Environment and Genes Related to Diseases (Xi’an Jiaotong University), Ministry of Education, Xi’an 710061, China; 2 Department of Cardiovascular Surgery, Xijing Hospital, Fourth Military Medical University, Xi’an 710032, China; 3 Department of Endocrinology and Metabolism, The First Affiliated Hospital of Xi’an Jiaotong University, Xi’an Jiaotong University Health Science Center, Xi’an 710061, China; 4 Key Laboratory of Cardiovascular Disease and Molecular Intervention, Department of Physiology, Nanjing Medical University, Nanjing 210029, China; 5 Department of Pharmacology, School of Basic Medical Sciences, Xi’an Jiaotong University Health Science Center, Xi’an 710061, China

## Abstract

Brain renin-angiotensin system (RAS) could regulate oxidative stress in the paraventricular nucleus (PVN) in the development of hypertension. This study was designed to explore the precise mechanisms of RAS acting on reactive oxygen species (ROS) in salt-induced hypertension. Male Wistar rats were administered with a high-salt diet (HS, 8.0% NaCl) for 8 weeks to induced hypertension. Those rats were received PVN infusion of AT1R antagonist losartan (LOS, 10 μg/h) or microinjection of small interfering RNAs for protein kinase C γ (PKCγ siRNA) once a day for 2 weeks. High salt intake resulted in higher levels of AT1R, PKCγ, Rac1 activity, superoxide and malondialdehyde (MDA) activity, but lower levels of copper/zinc superoxide dismutase (Cu/Zn-SOD), superoxide dismutase (SOD) and glutathione (GSH) in PVN than control animals. PVN infusion of LOS not only attenuated the PVN levels of AT1R, PKCγ, Rac1 activity, superoxide and decreased the arterial pressure, but also increased the PVN antioxidant capacity in hypertension. PVN microinjection of PKCγ siRNA had the same effect on LOS above responses to hypertension but no effect on PVN level of AT1R. These results, for the first time, identified that the precise signaling pathway of RAS regulating ROS in PVN is *via* AT1R/PKCγ/Rac1 in salt-induced hypertension.

High salt intake is a significant environmental factor, which strongly associated with blood pressure and the effects of salt loading on the renin-angiotensin system (RAS) in development of salt-induced hypertension^1^,^2^. It is reported that high salt intake elevated the concentration of [Na^+^] in cerebrospinal fluid (CSF) and led to the activation of brain RAS and the increase of angiotensin II (Ang II) production in Dahl sensitive rat^1^,^3^. As the primary effector peptide of the RAS, Ang II binding to the angiotensin II type-1 receptor (AT1R) contributes to the regulation of blood pressure and the development and/or the maintenance of metabolic syndrome^4^.

It is well known that paraventricular nucleus of the hypothalamus (PVN) in the brain is an important central site for integration of sympathetic nerve activity and regulation of cardiovascular function^5^,^6^. Abundant evidences suggest that the elevated AT1R leds to reactive oxygen species (ROS) accumulation such as superoxide radical, hydroxyl ion, and hydrogen peroxide in the PVN, which contribute to the overactivity of pre-autonomic PVN neurons and result in sympathoexcitation and metabolic disorders in the pathophysiology of hypertension^5^,^7^,^8^,^9^. Furthermore, data from our laboratory suggest that Ang II increased the PVN levels of ROS, and bilateral PVN infusion of AT1R blocker decreased the production of ROS in PVN, as well as sympathetic nerve activity and blood pressure^10^,^11^,^12^. Therefore, the activation of AT1R augments the intracellular level of ROS and sympathetic nerve activity. However, the precise signaling pathway of RAS modulating the oxidative stress in PVN is still unknown in hypertension.

Superoxide generated by NAD(P)H oxidase has emerged as a key intermediary in the central and peripheral effects of Ang II^13^. NAD(P)H oxidase is composed of membrane-bound subunits, gp91^phox^ and p22^phox^, cytoplasmic subunits, p47^phox^, p40^phox^, p67^phox^, and Rac1 and/or Rac2^14^. Rac1 (Ras-related C3 botulinum toxin substrate1) is a small GTPase which is essential for the assembly and activation of NAD(P)H oxidase and the production of superoxide^15^. A growing body of evidence indicate that the increased Ang II in brain induces the NAD(P)H-dependent ROS production by the protein kinase C (PKC) which is a critical step in Rac1 activation and subsequent enzyme assembly^13^,^16^. PKC is a family of phospholipids serine/threonine protein kinases, which have been classified into conventional PKCs (α, β and γ), novel PKCs (δ, ε, η and θ), and atypical PKCs (ζ and λ/ι)^17^. PKCγ, unlike other PKC isozymes, is expressed only in the central nervous system and eye tissues^17^. Aronowski and colleagues (2000) found that PKCγ is activated in the period of brain ischemia/reperfusion which is closely related to the oxidative stress^18^,^19^. These findings suggest that the linking of AT1R and NAD(P)H oxidase-derived ROS is involved in the effects of PKCγ/Rac1/NAD(P)H pathway. Furthermore, it is unclear whether AT1R/PKCγ/Rac1/NAD(P)H pathway in PVN mediates ROS production in the development of hypertension. Therefore, the aim of the present study was to determine whether the precise mechanism of RAS modulating oxidative stress in PVN is through AT1R/PKCγ/Rac1/NAD(P)H signaling pathway during the development of high-salt hypertension.

## Results

### Effect of losartan on mean arterial pressure (MAP)

**Figure 1:**
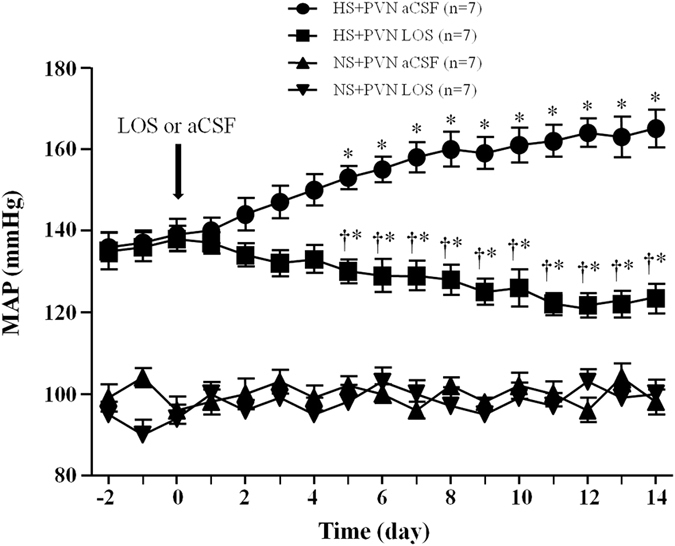
Effects of AT1R antagonist (LOS) on mean arterial pressure (MAP) in salt-induced hypertensive rats. MAP is increased in high salt (HS) intake rats. Bilateral PVN infusion of LOS for 14 days attenuated high salt-induced presser response on the 5^th^ day. Values are expressed as means ± SE. *P < *0.05 vs* NS groups (NS + PVN LOS or NS + aCSF); ^†^*P* < *0.05* HS + LOS *vs* HS + aCSF.

High salt diet induced a significant increase in mean arterial pressure (MAP) compared with control rats after 8 weeks (139 ± 4.7 mmHg *vs.* 94.7 ± 3.6 mmHg, P < 0.05) of high salt diet before bilateral PVN infusion of LOS or microinjections of PKCγ siRNA. In Fig. 1, HS + PVN aCSF exhibited a significant increase in MAP when compared with Wistar normal salt diet (NS) rats (at Day 14, 163.3 ± 5.6 *vs.* 95.6 ± 3.0 mmHg, *P* < *0.05*). Whereas HS + PVN LOS rats exhibited significantly reduced MAP from Day 5, and it remained lower for the duration of the study (at Day 14, 119.2 ± 3.5 *vs.* 165.4 ± 5.3 mmHg, *P* < *0.05*) when compared with HS + PVN aCSF rats.

### Effect of losartan on sympathetic nerve activity

**Figure 2:**
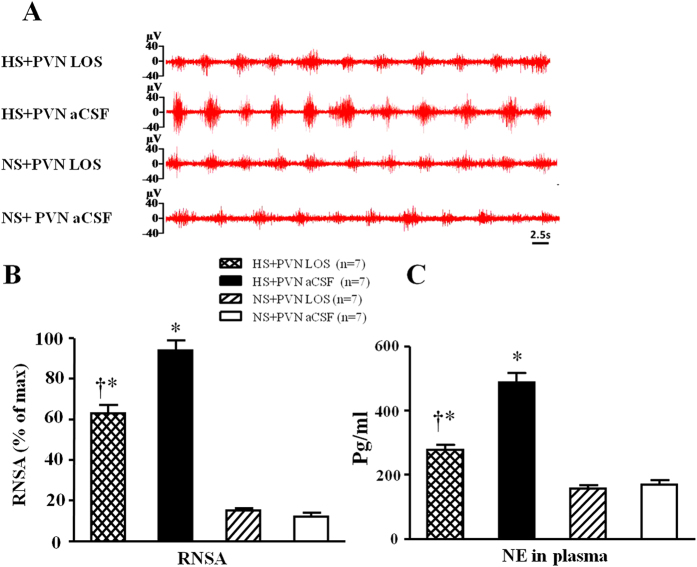
Effects of LOS on renal sympathetic nerve activity (RSNA) and norephedrine (NE) in plasma in salt-induced hypertensive rats. RSNA was increased in high salt (HS) intake rats. Bilateral PVN infusion of LOS decreased RSNA in HS rats. (**A**) A representative renal sympathetic nerve activity in different groups. (**B**) Bar graph comparing renal sympathetic nerve activity in different groups. (**C**) Bar graph comparing NE in plasma in different groups. Values are expressed as means ± SE. *P < *0.05 vs* NS groups (NS + PVN LOS or NS + aCSF); ^†^*P* < *0.05* HS + LOS *vs* HS + aCSF.

In Fig. 2, HS + PVN aCSF rats exhibited a 7.1-fold increase in renal sympathetic nerve activity (RSNA, % of max) and 3.3-fold increase in plasma noradrenaline (NE) when compared with control rats (P < 0.05). Bilateral PVN infusion of LOS declined to a 64.5% decrease RSNA and 54.7% plasma NE in high salt-induced hypertensive rats (P < 0.05). NS + PVN LOS did not attenuate RSNA when compared with NS + PVN aCSF.

### Effect of losartan on PKCγ and AT1R positive neurons in PVN

**Figure 3:**
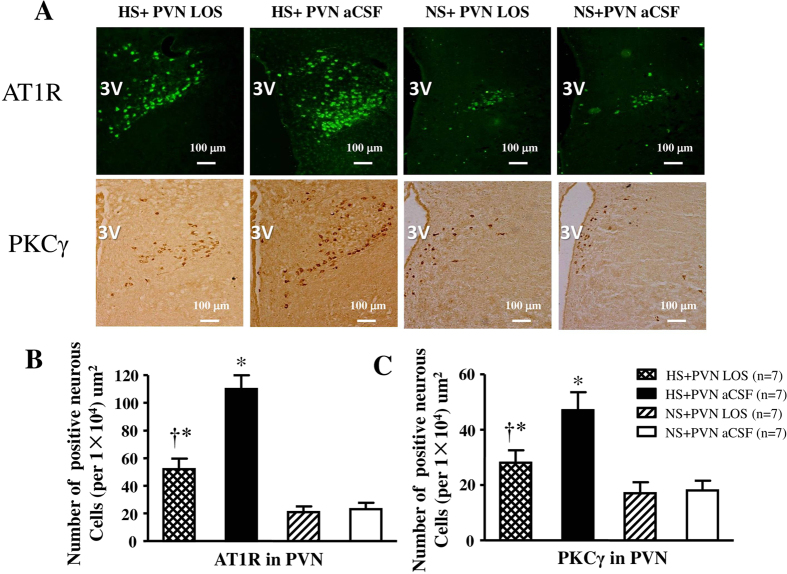
Effects of LOS on AT1R and PKCγ expression in the PVN of salt-induced hypertensive rats. Expressions of AT1R and PKCγ in high salt intake (HS) rats were higher than in control rats. Treatment with LOS attenuated the AT1R and PKCγ expression in the PVN of HS rats. (**A**) Immunofluorescence for AT1R (bright green) and immunohistochemistry for PKCγ (bright brown) in the PVN in different groups. (**B**) and (**C**) Column diagram showing the numbers of AT1R and PKCγ positive neurous cells in the PVN in different groups. *P < *0.05 vs* NS groups (NS + PVN LOS or NS + aCSF); ^†^*P* < *0.05* HS + LOS *vs* HS + aCSF.

In Fig. 3, immunohistochemistry and immunofluorescence results revealed that high salt diet respectively induced a 4.8-fold increase and 2.6-fold increase in the number of AT1R and PKC**γ** positive neurons compared with control rats (P < 0.05). Bilateral PVN infusion of LOS respectively declined to a 51.2% decrease and 60% decrease in the number of AT1R and PKC**γ** positive neurons in hypertensive rats (P < 0.05). NS + PVN LOS did not attenuate the number of AT1R and PKC**γ** positive neurons when compared with NS + PVN aCSF.

### Effect of losartan on p-Rac1 positive neurons and the level of superoxide in PVN

**Figure 4:**
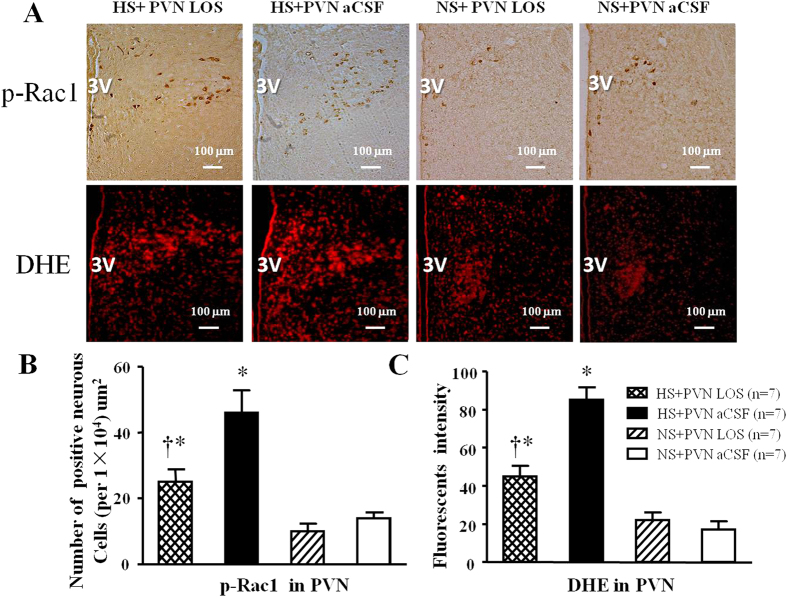
Effects of LOS on p-Rac1 expression and ROS activity in the PVN of salt-induced hypertensive rats. ROS activity was measured by fluorescent-labeled dihydroethidium (DHE) staing. High salt (HS) intake rats had higher level of p-Rac1 expression and immunofluorescent intensity of DHE when compared with control rats. Bilateral PVN infusion of LOS for14 days reduced the levels of superoxide and p-Rac1 expression in the PVN of HS rats. (**A**) Immunohistochemistry for p-Rac1 and the immunofluorescence for superoxide in the PVN in different groups. (**B**) Column diagram showing the effects of LOS on the p-Rac1 positive neurons in the PVN in different groups. (**C**) Column diagram showing immunofluorescent intensity of DHE in the PVN in different groups. Values are expressed as means ± SE. *P < *0.05 vs* NS groups (NS + PVN LOS or NS + aCSF); ^†^*P* < *0.05* HS + LOS *vs* HS + aCSF.

In Fig. 4, immunohistochemistry and immunofluorescence results revealed that high salt diet respectively induced a 2.3-fold increase and 3.5-fold increase in the number of p-Rac1 positive neurons and superoxide compared with control rats (P < 0.05). Bilateral PVN infusion of LOS respectively declined to a 56.0% decrease and 53.1% decrease in the number of p-Rac1 positive neurons and superoxide in hypertensive rats (P < 0.05). NS + PVN LOS did not attenuate the number of p-Rac1 positive neurons and superoxide when compared with NS + PVN aCSF.

### Effect of losartan on protein expression levels of AT1R, PKCγ, p-Rac1 and Cu/Zn-SOD in PVN

**Figure 5:**
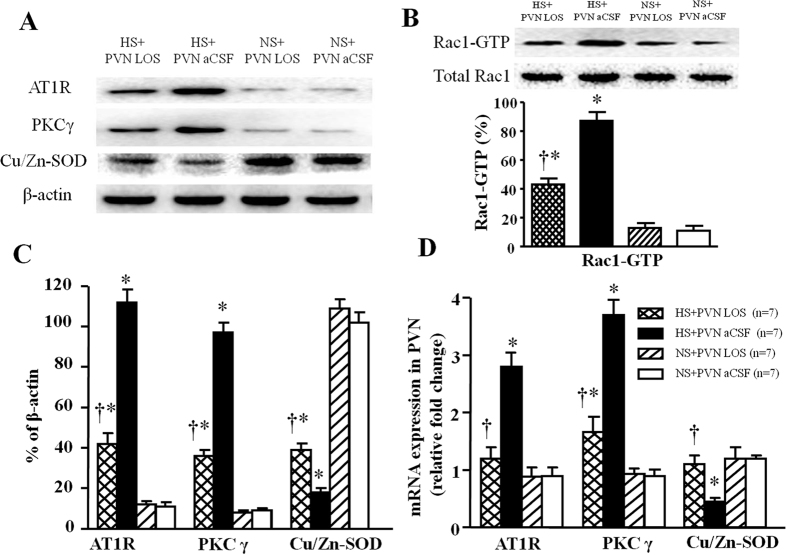
Effects of LOS on AT1R, PKCγ, GTP-Rac1 (Pull-down Assay) and Cu/Zn-SOD proteins expression, and effects of LOS on AT1R, PKCγ and Cu/Zn-SOD mRNA level in the PVN of salt-induced hypertensive rats. High salt (HS) intake rats had higher levels of AT1R, PKCγ and GTP-Rac1, but lower level of Cu/Zn-SOD compare with control rats. Bilateral PVN infusion of LOS for 14 days attenuated expression of AT1R, PKCγ and GTP-Rac1, and augmented expression of Cu/Zn-SOD in hypertensive rats. (**A**) A representative immunoblot; (**B**) PVN tissues were analyzed for Rac1 activity (Rac1-GTP) as described in METHODS. As controls, protein levels of Rac1 (total-Rac1) in PVN were measured. (**C**) Densitometric analysis of protein expression of AT1R, PKCγ and Cu/Zn-SOD in the PVN in different groups; (**D**) Densitometric analysis of mRNA expression of AT1R, PKCγ and Cu/Zn-SOD in the PVN in different groups. Values are expressed as means ± SE. *P < *0.05 vs* NS groups (NS + PVN LOS or NS + aCSF); ^†^*P* < *0.05* HS + LOS *vs* HS + aCSF.

In Fig. 5A–C, western blot results indicated that high salt-induced rats respectively induced a 10.3-fold (AT1R), 8.9-fold (PKCγ) and 9.7-fold (GTP-Rac1, Pull-down Assay) increase, but 5.6-fold decrease (Cu/Zn-SOD) in the protein expression level (P < 0.05) in PVN compared with control rats. Bilateral PVN infusion of LOS respectively declined to 38% (AT1R), 37% (PKCγ), and 64% (GTP-Rac1, Pull-down Assay) protein expression level, but increased Cu/Zn-SOD protein level to 46% compared with high salt-induced rats (P < 0.05).

### Effect of losartan on mRNA levels of AT1R, PKCγ, and Cu/Zn-SOD in PVN

In Fig. 5D, the high salt diet respectively induced a 3.1-fold (AT1R) and 4.2- fold (PKCγ) but 3.0-fold decrease (Cu/Zn-SOD) in the mRNA level in PVN compared with control rats (P < 0.05). Bilateral PVN infusion of LOS respectively declined to 42.8% (AT1R) and 43.2% (PKCγ) mRNA level, but increased Cu/Zn-SOD mRNA level to 36.5% compared with high salt-induced rats (P < 0.05).

### Effect of PKCγ (PKCγ siRNA) on mean arterial pressure (MAP)

**Figure 6:**
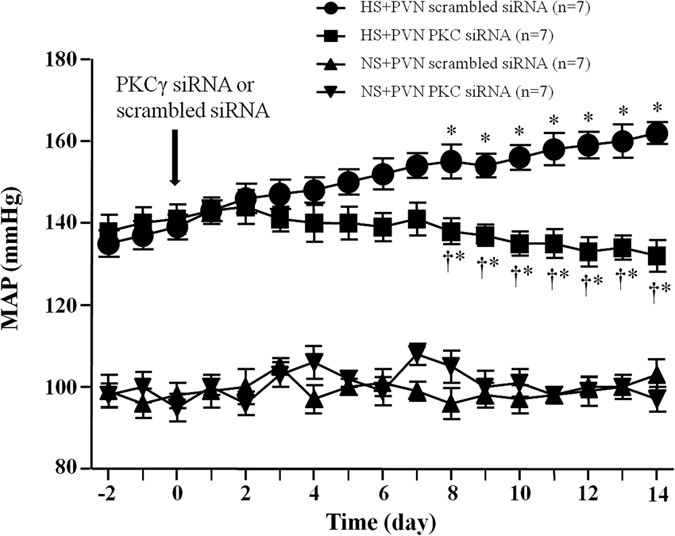
Effects of PKCγ siRNA on mean arterial pressure (MAP) in salt-induced hypertensive rats. MAP is increased in high salt (HS) intake rats. Bilateral PVN microinjections of PKCγ for 14 days attenuated high salt-induced presser response on the 8^th^ day. Values are expressed as means ± SE. *P < *0.05 vs* NS groups (NS + PKCγ siRNA or NS + scrambled siRNA); ^†^*P* < *0.05* HS + PKCγ siRNA *vs* HS + scrambled siRNA.

In Fig. 6, the MAP of HS + PVN scrambled siRNA group is also much higher than control animals (at Day 14, 160.6 ± 4.9 *vs.* 95.1 ± 3.1 mmHg, *P* < *0.05*) at the end of experiment. Bilateral PVN microinjection of PKCγ siRNA significantly decreased the MAP compared to HS + PVN scrambled siRNA rat (at Day 14, 132.6 ± 3.4 *vs.* 163.5 ± 5.6 mmHg, *P* < *0.05*). Normal salt diet groups did not show any significant change in MAP at the end of experiment.

### Effect of PKCγ (PKCγ siRNA) on sympathetic nerve activity

**Figure 7:**
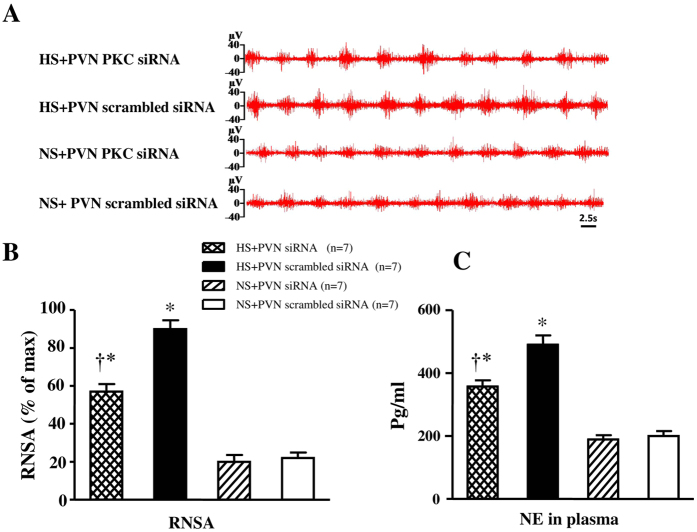
Effects of PKCγ siRNA on RSNA and NE in plasma in salt-induced hypertensive rats. RSNA was increased in high salt (HS) intake rats. Bilateral PVN infusion of PKCγ siRNA decreased RSNA in HS rats. (**A**) A representative renal sympathetic nerve activity in different groups. (**B**) Bar graph comparing renal sympathetic nerve activity in different groups. (**C**) Bar graph comparing NE in plasma in different groups. Values are expressed as means ± SE. *P < *0.05 vs* NS groups (NS + PKCγ siRNA or NS + scrambled siRNA); ^†^*P* < *0.05* HS + PKCγ siRNA *vs* HS + scrambled siRNA.

In Fig. 7, HS + PVN scrambled siRNA rats exhibited a 4.1-fold increase in RSNA and a 2.5-fold increase in plasma NE when compared with control rats (P < 0.05). Bilateral PVN infusion of PKCγ siRNA declined to a 65.4% RSNA and 72% plasma NE in high salt-induced hypertensive rats (P < 0.05). NS + PVN PKCγ siRNA did not show any significant change in RSNA and plasma NE when compared with NS + PVN scrambled siRNA at the end of experiment.

### Effect of PKCγ (PKCγ siRNA) on PKCγ and AT1R positive neurons in PVN

**Figure 8:**
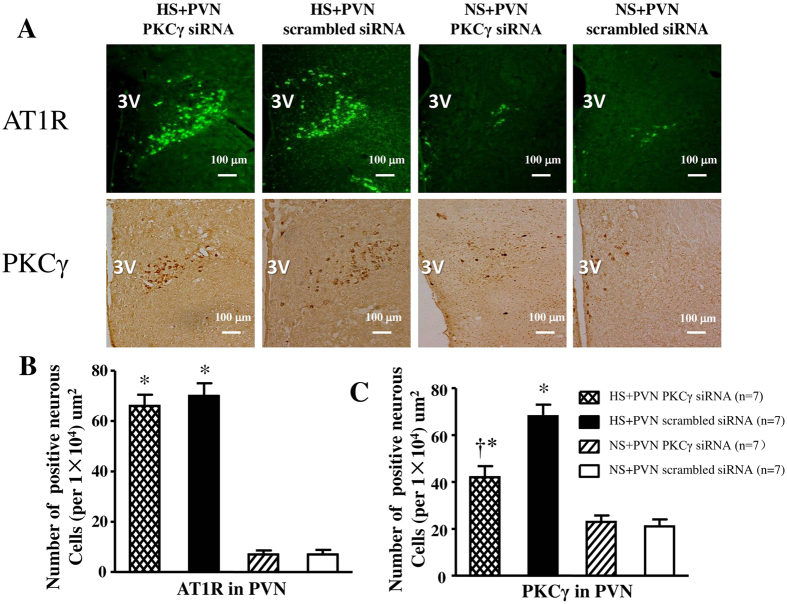
Effects of PKCγ siRNA on AT1R and PKCγ expression in the PVN of salt-induced hypertensive rats. Expressions of AT1R and PKCγ in high salt intake (HS) rats were higher than in control rats. Microinjections of PKCγ siRNA attenuated the PKCγ expression and had no effect on the AT1R positive neurons in the PVN of HS rats. (**A**) Immunofluorescence for AT1R (bright green) and immunohistochemistry for PKCγ (bright brown) in the PVN in different groups. (**B**) and (**C**) Column diagram showing the numbers of AT1R and PKCγ positive neurous cells in the PVN in different groups. Values are expressed as means ± SE. *P < *0.05 vs* NS groups (NS + PKCγ siRNA or NS + scrambled siRNA); ^†^*P* < *0.05* HS + PKCγ siRNA *vs* HS + scrambled siRNA.

In Fig. 8, high salt diet also induced respectively induced a 6.7-fold increase and 3.2-fold increase in the number of AT1R and PKC**γ** positive neurons compared with control rats (P < 0.05). However, bilateral PVN microinjection of PKCγ siRNA declined to 60% PKCγ positive neurons, and had no effect on the AT1R positive neurons in PVN in hypertensive rats at the end of experiment.

### Effect of PKCγ (PKCγ siRNA) on p-Rac1 positive neurons and the level of Superoxide in PVN

**Figure 9:**
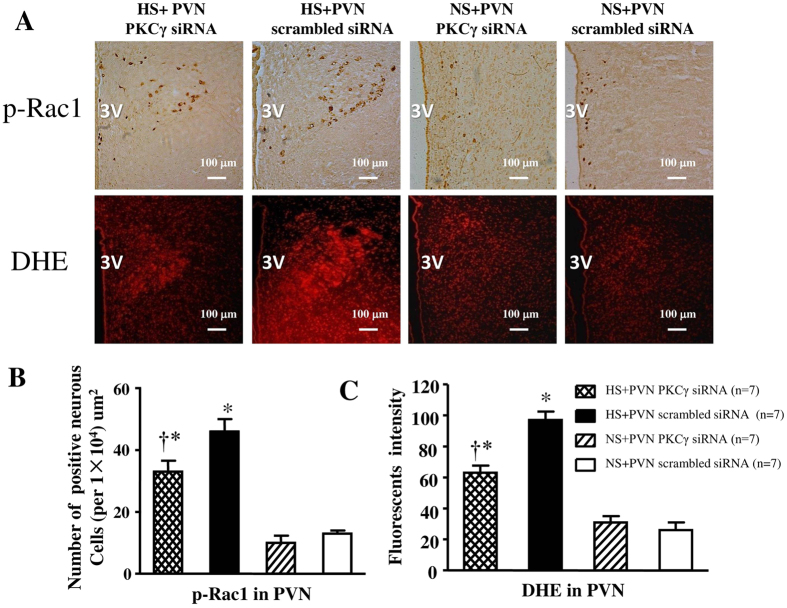
Effects of PKCγ siRNA on p-Rac1 expression and ROS activity in the PVN of salt-induced hypertensive rats. ROS activity was measured by fluorescent-labeled dihydroethidium (DHE) staing. High salt (HS) intake rats had higher level of p-Rac1 expression and immunofluorescent intensity of DHE when compared with control rats. Bilateral PVN microinjections of PKCγ siRNA for14 days reduced the levels of superoxide and p-Rac1 expression in the PVN of HS rats. (**A**) Immunohistochemistry for p-Rac1 and the immunofluorescence for superoxide in the PVN in different groups. (**B**) Column diagram showing the effects of PKCγ siRNA on the p-Rac1 positive neurons in the PVN in different groups. (**C**) Column diagram showing immunofluorescent intensity of DHE in the PVN in different groups. Values are expressed as means ± SE. *P < *0.05 vs* NS groups (NS + PKCγ siRNA or NS + scrambled siRNA); ^†^*P* < *0.05* HS + PKCγ siRNA *vs* HS + scrambled siRNA.

In Fig. 9, high salt diet also induced respectively induced a 1.3-fold increase and 3.7-fold increase in the number of p-Rac1 positive neurons and superoxide compared with control rats (P < 0.05). However, bilateral PVN microinjection of PKCγ siRNA respectively declined to 34% the number of p-Rac1 positive neurons and 70% superoxide in PVN in hypertensive rats at the end of experiment (P < 0.05).

### Effect of PKCγ (PKCγ siRNA) on protein expression levels of AT1R, PKCγ, p-Rac1 and Cu/Zn-SOD in PVN

**Figure 10:**
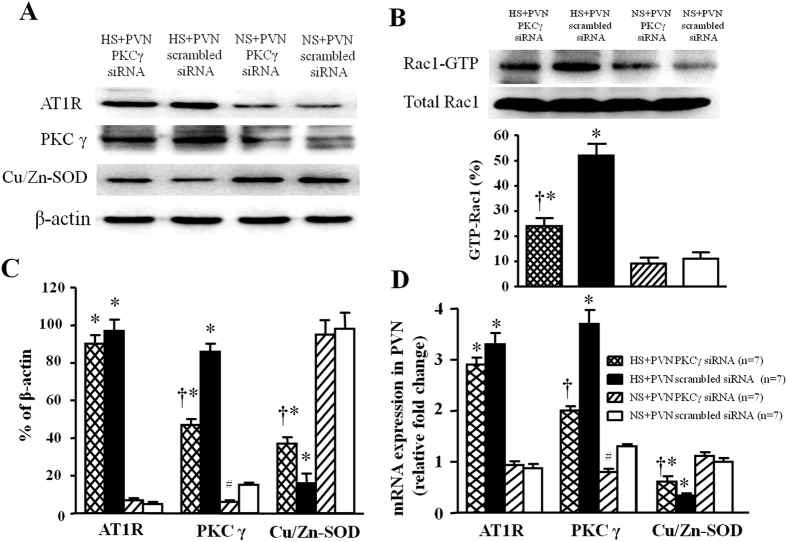
Effects of PKCγ siRNA on AT1R, PKCγ, GTP-Rac1 (Pull-down Assay) and Cu/Zn-SOD proteins expression, and effects of LOS on AT1R, PKCγ and Cu/Zn-SOD mRNA level in the PVN of salt-induced hypertensive rats. High salt (HS) intake rats had higher levels of AT1R, PKCγ and GTP-Rac1, but lower level of Cu/Zn-SOD compare with control rats. Bilateral PVN infusion of PKCγ siRNA for 14 days attenuated expression of AT1R, PKCγ and GTP-Rac1, and augmented expression of Cu/Zn-SOD in hypertensive rats. However, admiration of PKCγ siRNA in PVN had no effect on PVN levels of AT1R in salt-induced hypertension. (**A**) A representative immunoblot; (**B**) PVN tissues were analyzed for Rac1 activity (Rac1-GTP) as described in METHODS. As controls, protein levels of Rac1 (total-Rac1) in PVN were measured. (**C**)densitometric analysis of protein expression of AT1R, PKCγ and Cu/Zn-SOD in the PVN in different groups; (**D**) Densitometric analysis of mRNA expression of AT1R, PKCγ and Cu/Zn-SOD in the PVN in different groups. Values are expressed as means ± SE. *P < *0.05 vs* NS groups (NS + PKCγ siRNA or NS + scrambled siRNA); ^†^*P* < *0.05* HS + PKCγ siRNA *vs* HS + scrambled siRNA. ^#^*P* < *0.05* NS + PKCγ siRNA *vs* NS + scrambled siRNA.

In Fig. 10A–C, western blot results indicated that high salt-induced rats respectively induced a 9.6-fold (AT1R), 1.8-fold (PKCγ) and 7.3-fold (GTP-Rac1, Pull-down Assay) increase, but 7.8-fold decrease (Cu/Zn-SOD) in the protein expression level (P < 0.05) in PVN compared with control rats. Bilateral PVN infusion of PKCγ siRNA in PVN respectively declined to 51% (AT1R), 31% (PKCγ), and 49% (GTP-Rac1, Pull-down Assay) protein expression level, but increased Cu/Zn-SOD protein level to 32% compared with high salt-induced rats (P < 0.05). And administration of PKCγ siRNA in PVN had no effect on PVN levels of AT1R in salt-induced hypertensive rats. Bilateral PVN infusion of PKCγ siRNA in normal rat downregulated a 55% protein expression compared with normal rats with scrambled siRNA.

### Effect o PKCγ (PKCγ siRNA) on mRNA levels of AT1R, PKCγ, and Cu/Zn-SOD in PVN

In Fig. 10D, RT-PCR results indicated that high salt-induced rats respectively induced a 10.1-fold (AT1R) and 8.4-fold (PKCγ), but 7.2-fold decrease (Cu/Zn-SOD) in the protein expression level (P < 0.05) in PVN compared with control rats. Bilateral PVN infusion of PKCγ siRNA in PVN respectively declined to 31% (PKCγ) mRNA level, but increased Cu/Zn-SOD mRNA level to 36% compared with high salt-induced rats (P < 0.05). And administration of PKCγ siRNA in PVN had no effect on PVN mRNA levels of AT1R in salt-induced hypertensive rats. Bilateral PVN infusion of PKCγ siRNA in normal rat downregulated a 52% mRNA level compared with normal rats with scrambled siRNA.

### Effect of losartan and PKCγ (PKCγ siRNA) on arterial pressure and heart rate (HR)

The mean arterial pressure (MAP) and heart rate (HR) were measured with a pressure transducer (MLT0380, AD Instruments, Australia) *via* a catheter in the right carotid artery. The MAP and HR in high salt-induced hypertensive rats were significantly higher than that in normal salt diet rats. There was no significant difference in the body weight between hypertensive rats and control rats. Chronic PVN infusion of LOS and PKCγ siRNA attenuated MAP and HR in hypertensive rats, but not in normal salt diet rats (Table 1).

### Effect of losartan and PKCγ (PKCγ siRNA) on oxidative stress in PVN

Compared with NS rats, HS + PVN aCSF and HS + PVN scrambled siRNA groups had higher level of malondialdehyde (MAD) and NAD(P)H oxidase activity, but lower levels of superoxide dismutase (SOD) and glutathione (GSH) in PVN. Infusion of LOS or microinjection of PKCγ siRNA into the PVN attenuated the increased levels of MAD and NAD(P)H oxidase activity, but decreased levels of SOD activity and GSH in PVN of HS groups (Table 2).

## Discussion

The novel finding of this study is that both AT1R and PKCγ play an important role in modulating the salt-induced central RAS activation and oxidative stress responses. And these results, for the first time, identified that the precise signaling pathway of RAS regulating ROS in PVN may be through AT1R/PKCγ/Rac1 pathway, which induces overproduction of ROS from Rac1-dependent NAD(P)H and finally increase sympathetic nerve activity and blood pressure. Therefore, PKCγ signaling pathway contributes to RAS modulating oxidative stress in PVN in the development of salt-induced hypertension.

PVN is a central integration site for the regulation of cardiovascular functions^2^. Substantial findings have confirmed high salt intake is a significant environmental factor, which strongly associated with RAS and ROS in the PVN^3^,^20^. Our study also showed that the activated RAS could regulate the reactive oxygen species overproduction in PVN in the progression of hypertension. As the primary effector peptide of the RAS, Ang II binding to AT1R contributes to the regulation of blood pressure and the development and/or the maintenance of metabolic syndrome. There is also increasing evidence showed that the augmented Ang II in brain induces the NAD(P)H-dependent ROS production by PKC. Members of the PKC family play a key role in the regulation of cellular functions in the nervous system^21^. Meanwhile, previous work by Griendling K and coworkers (2009) reviewed that Ang II activated by NAD(P)H oxidase is mainly dependent on the PKC and other signaling pathways in the central nervous system^13^. Lin D. and Takemoto D. J. (2005) had showed that PKCγ could activate the oxidative stress in the cardiovascular and neurodegenerative diseases^22^. And PKCγ is a unique isoform of PKC that is found in neurons and eye tissues. Rac1 (Ras-related C3 botulinum toxin substrate1) is a small GTPase which is essential for the assembly and activation of NAD(P)H oxidase^23^. Growing evidence indicate that NOX/Rac1 activation is a main pathway in cardiovascular and cerebrovascular disorders such as myocardial infarction, hypertension, atherosclerosis and stroke^14^. Zimmerman M. C. (2004) had, for the first time, identified a Rac1-dependent NAD(P)H oxidase as the source of central Ang II-induced O_2_ production, and implicated this oxidase in cardiovascular diseases associated with dysregulation of brain Ang II signaling, including hypertension^14^. Moreover, increasing evidences demonstrate that increased Ang II in brain induces the NAD(P)H-dependent ROS production by PKC is a critical step in Rac1 activation and subsequent enzyme assembly.

**Figure 11:**
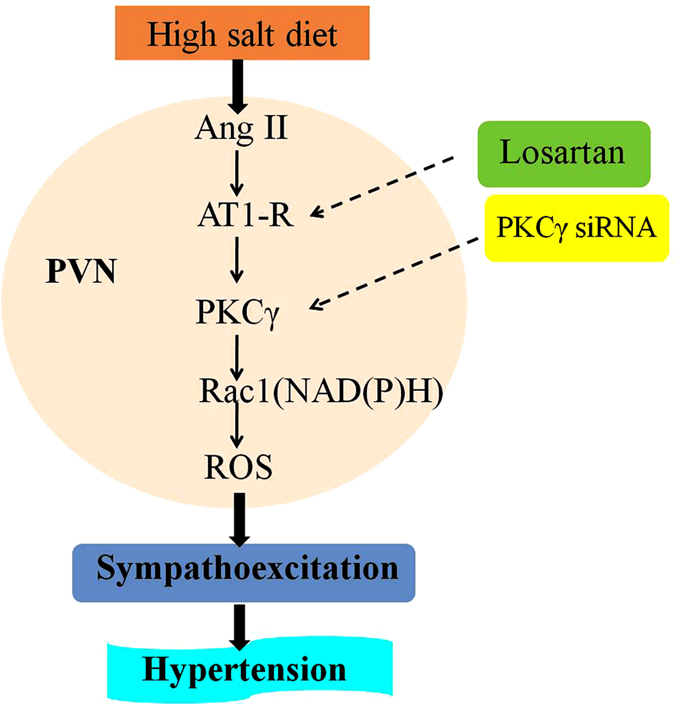
The schematic showing the proposed signaling pathways of renin-angiotensin system (RAS) regulating reactive oxygen species (ROS) within the hypothalamic paraventricular nucleus (PVN) *via* AT1R/PKC γ/Rac1 in high salt-induced hypertension.

In this study, PVN microinjection of PKCγ siRNA could decrease the sympathetic nervous activity and mean arterial pressure, which indicates that PKCγ plays a significant role in the brain Ang II regulation of oxidative stress in the pathophysiology of high salt-induced hypertension. And our results also show that high salt diet not only elevated the PVN level of AT1R, PKCγ, Rac1, and NADPH oxidase-dependent superoxide in PVN, but also decreased the antioxidant capacity in PVN, therefore, increased the sympathetic nervous activity and mean arterial pressure. Chronic infusion of AT1R antagonist (LOS) into PVN decreased the PVN expression of AT1R, PKCγ, Rac1 and the level of superoxide in hypertensive rats, but increased the antioxidant capacity in PVN. Campese VM (2000) found LOS could stimulate the production of nitric oxide in PVN, and the nitric oxide could regulate the sympathetic outflow and reduces blood pressure levels^24^. This response is also consistent with our results. However, PVN microinjection of PKCγ siRNA suppressed NAD(P)H oxidase-dependent oxidative stress in PVN, decreased the PVN levels of PKCγ, Rac1 and NAD(P)H oxidase subunits but did not affect AT1R. So we would only speculate that AT1R could act on PKCγ and Rac1-dependent NAD(P)H oxidase and regulates the NAD(P)H oxidase-dependent oxidative stress in PVN, which implicated RAS regulating ROS in PVN may be through the AT1R/PKCγ/Rac1 signaling pathway and increase the sympathetic nervous activity and mean arterial pressure in the progression of salt-induced hypertension, which is presented in Fig. 11.

## Material and Methods

### Animals

Male Wistar rats weighing 150 g to 200 g were purchased from Experimental Animal Center of Xi’an Jiaotong University. All animals were housed in temperature (23 ± 2 °C) and light-controlled (12 h light/dark cycle) animal quarters and were provided with rat chow *ad libitum*. All experimental procedures were reviewed and approved by the National Institutes of Health Guide for the Care and Use of Laboratory Animals (the US National Institutes of Health Publication No. 85–23, revised 1996). All of the animal procedures were conducted according to the Animal Care and Use Committees of Xi’an Jiaotong University.

### PKC λ siRNA preparation and transfection in PVN

PKCλ siRNA (siPKC) oligonucleotides and the scrambled siRNA were purchased from GenePharma (Shanghai, China). We selected three potential siRNA sequences with high knock-down probability values. The siRNA sequences in Prkcg-Rat started at bases 364, 1277, and 1537 (364: 5′-AAGUCGGUACAGUGACUGCTT-3′; 1277:5′-UUUCCCUAGAACCAUGAGGTT-3′; 1537:5′-AUGUGGUACAUUAAAUCGCTT-3′). To test the silence ability of these siRNA sequences target genes in the PVN, 15 nM of each siRNA carried out with Lipofectamine TM 2000 (Invitrogen) was infusion of PVN for two weeks in the high salt-induced hypertensive rats according to the manufacturer’s instructions. After 14 days, the rats were euthanized to collect PVN tissue. Western blot was used for measurement of PKC proteins expression in PVN. Finally, we choose PKCγ siRNA sequences 1537 as follows: 5′-AUGUGGUACAUUAAAUCGCTT-3′^2^ in our further study.

### General experimental protocol

Rats were fed on the normal salt diet containing 0.3% NaCl (NS) or high salt diet containing 8% NaCl (HS) for 8 weeks to induce hypertension. After 8 weeks, animals from NS group and HS group are received bilateral PVN infusion of AT1R anantagonist losartan (LOS, 10 μg/h) or artificial cerebrospinal fluid (aCSF), and bilateral PVN microinjection of PKCγ small interfering RNA (PKCγ siRNA), or scrambled siRNA for 2 weeks with high salt diet respectively^25^.

### Implantation of bilateral PVN osmotic minipump for chronic infusion

The method for PVN minipump has been previously described^8^,^26^,^27^. Each rat head was placed into a stereotaxic apparatus after anesthetized with ketamine (90 mg/kg) and xylazine (10 mg/kg) intraperitoneally (i.p). A skin incision was made, and the skull was opened. The location was 1.8 mm caudal to the bregma, 0.4 mm lateral to central line, and 7.9 mm below the skull surface (Chen *et al*., 2011; Zhu *et al*., 2004). The skull was then opened, and the minipumps (ALZET Osmotic Pumps, 2002 Model, 0.5 ul/h) connected with losartan (LOS, 10 μg/h) or artificial cerebrospinal fluid (aCSF) for the continuous infusion were implanted subcutaneously in the back of the neck. The infusion was continued for 2 weeks. Rats received buprenorphine (0.01 mg/kg, sc) immediately following surgery. The success rate of bilateral PVN microinjection was around 68%.

### Bilateral PVN cannulae implantation for chronic infusion studies

The method for implantation of bilateral PVN cannulae has been described previously. Briefly, under anesthesia, rat head was placed into a stereotaxic apparatus. A stainless steel double cannula (Plastics One, Inc.) with a center-to-center distance of 0.5 mm, was implanted into the PVN using an introducer, according to stereotaxic coordinates (1.8 mm caudal to the bregma, 0.4 mm lateral to central line, and 7.9 mm below the skull surface)^10^. The cannula was fixed to the cranium using dental acrylic and two stainless steel screws. 50 nL of scrambled siRNA or small interfering RNAs for PKCγ (PKCγ siRNA) were microinjected into the bilateral PVN each side, which were completed within 1 min once per day. The success rate of bilateral PVN cannulation is about 65%.

### Blood pressure measurement

Arterial pressure was measured noninvasively *via* tail-cuff instrument and their Recording System. Conscious rats from each group were warmed to an ambient temperature of 30 °C by placing them in a holding device mounted on a thermostatically controlled warming plate. Each rat was allowed to accommodate the cuff for 10 minutes before blood pressure measurement. The rat arterial pressure was measured every week during the 8 weeks and every day after LOS or siRNA infusion. Mean arterial pressure and heart rate data were consisted of 20 times, which were collected for 40 min within the same 2-hour time window each day and then averaged those data until the end of this study^28^.

### Sympathetic neural recordings

Rats were anaesthetized with a ketamine (90 mg/kg) and xylazine (10 mg/kg) mixture (ip). After retroperitoneal laparotomy, the left renal nerves were isolated *via* glass microelectrode technique under an inversion microscope. The renal nerve was hung by a platinum electrode which is connected to the recording system. In order to moisturize the nerves and isolate electrical disturbance, the nerve should be covered by paraffin oil tampons. Maximum renal sympathetic nerve activity (RSNA) was detected using an intravenous bolus administration of sodium nitroprusside (SNP, 10 mg). The recordings of rectified and integrated RSNA were analyzed using methods described as previously^8^,^26^.

### Immunohistochemistry and immunofluorescence staining

Rats were anaesthetized and received a thoracotomy and were perfused through the left ventricle first with 300 mL of 0.01 M phosphate-buffered solution (PBS) at pH 7.4 and then with 300 mL of 4% paraformaldehyde. The brains were immediately removed and immersed in 4% paraformaldehyde, and then immersed in 30% sucrose for at least 2 days. Samples were embedded in OCT and microdissection procedures were used to isolate the PVN tissue. The tissues were collected from both sides of the PVN of individual rat and sectioned into several 18 mm transverse sections at about 1.80 mm from bregma and stored at −80 °C for future use for immunohistochemistry and immunofluorescence staining^5^.

Immunohistochemistry and immunofluorescence were performed as described previously^2^. The primary antibodies (AT1R (sc-160811), PKCγ (sc-211), p-Rac1 (sc-12924-R), and Cu/Zn-SOD (sc-8637) were purchased from Santa Cruz Biotechnology. Superoxide anion levels in PVN were determined by fluorescent-labeled dihydroethidium (DHE; Molecular Probes) staining. Coronal sections (14 um) were incubated for 10 minutes with DHE (1 umol/L, Sigma) at 37 °C, as previously described^5^.

### Rac1 activity assay

Rac1 activity was assayed using a pull-down assay (Millipore kit, Cat. #17-283) as described previously^29^,^30^. Briefly, the PVN tissue lysate was prepared by homogenization in ice-cold 1 × lysis buffer (diluted from 5 × Mg^2+^ Lysis Buffer, MLB, Cat. #20-168, Millipore) and placed on ice for 30 min. After 30 min, the lysate was centrifuged at 14,000 g for 15 min and the supernatant was collected. The sample concentrations were determined using the BCA Protein Assay Kit (Beyotime Institute of Biotechnology). Protein p21-activated protein kinase 1 (Pak1) is an effect protein that can bind active Rac1. One mg of total protein was incubated with 10 ul PAK-1 PBD (a GST fusion protein corresponding to the p21-binding domain (PBD) of human PAK-1) agarose beads for 1 h; the beads were then washed 3 times. The agarose beads were resuspended in 40 uL of 2 × reducing sample buffer and boiled for 5 min. The bound proteins were separated by 12% SDS-PAGE. GTP-bound Rac1 was detected by immunoblotting with an anti-Rac1antibody (Cat. #05-389, Millipore).

### Western blotting

Rats were sacrificed and decapitated. The PVN were quickly collected on ice and then submerged in liquid nitrogen. Tissues were stored at −80 °C until further testing. Protein extracted from PVN tissue was used for measuring the expression. PVN tissue samples were lysed in a RIPA buffer with protease inhibitor and phosphatase inhibitor cocktail. Sonication the protein content of the resulting samples was determined by a modified BCA protein assay. The samples were then subjected to sodium dodecyl sulfate-polyacrylamide gel electrophoresis using Electrophoresis and Blotting Apparatus (Bio-Rad). Protein products were separated by SDS-PAGE electrophoresis and transferred to nitrocellulose membranes. Then membranes were blocked with 3.0% BSA in TBST buffer (TBS plus 0.1% Tween-20) for 1.5 h at room temperature and incubated with primary antibodies overnight at 4 °C.

The primary antibodies for AT1R (sc-160811), PKCγ (sc-211), and Cu/Zn-SOD (sc-8637) were from Santa Cruz Biotechnology. Protein loading was controlled by probing all western blots with β-actin antibody which was from Santa Cruz Biotechnology and normalizing AT1R, PKCγ, and Cu/Zn-SOD protein intensities to that of β-actin. Band densities were analyzed using NIH Image J software^12^.

### Real-time PCR

The hypothalamic tissue containing PVN was dissected as described previously. Briefly, rat brains were isolated and cut into a coronal segment (−0.92 mm to −2.13 mm posterior to bregma). A block of the hypothalamus containing PVN was excised from the coronal section. Total RNA was extracted from microdissected PVN using Tri-Zol reagent (Invitrogen) and reverse transcribed using oligo (dT) with conditions at 23 °C for 10 min, 37 °C for 60 min, and 95 °C for 5 min. The cDNA used for real-time PCR with specific primers for AT1R, PKCγ and Cu/Zn-SOD and GAPDH were shown in Table 3. The quantitative fold changes in mRNA expression were determined relative to glyceraldehyde-phosphate dehydrogenase (GAPDH) mRNA levels in each corresponding group^6^,^15^.

### ELISA studies

MDA, SOD, GSH and NAD(P)H oxidase activity in PVN were quantified using commercially available rat ELISA kits (Invitrogen Corporation, CA, USA) according to the manufacturer’s instructions^29^,^30^,^31^,^32^. NE in plasma were quantified using commercially available rat ELISA kits (Invitrogen Corporation, CA, USA) according to the manufacturer’s instructions^6^.

### Statistical analysis

All data are expressed as mean ± SE. The significance of differences between mean values was analyzed by ANOVA followed by Tukey’test. A probability value of P < 0.05 was considered to be statistically significant.

## Additional Information

**How to cite this article**: Su, Q. *et al*. Renin-angiotensin system acting on reactive oxygen species in paraventricular nucleus induces sympathetic activation *via* AT1R/PKCγ/Rac1 pathway in salt-induced hypertension. *Sci. Rep.* **7**, 43107; doi: 10.1038/srep43107 (2017).

**Publisher's note:** Springer Nature remains neutral with regard to jurisdictional claims in published maps and institutional affiliations.

## Figures and Tables

**Table 1:** Changes of body weight, MAP and HR at the end of the 2nd week of the experiment (n = 7, for each group).

PVN	Body weight (g)	MAP (mmHg)	HR (bpm)
HS + PVN LOS	367 ± 9	119 ± 5^†,∗^	342 ± 10^†^
HS + PVN aCSF	355 ± 7	165 ± 8^∗^	384 ± 13^∗^
NS + PVN LOS	359 ± 8	100 ± 6	354 ± 8
NE + PVN aCSF	347 ± 8	98 ± 4	346 ± 9
HS + PVN PKC siRNA	358 ± 9	132 ± 7^†,∗^	351 ± 11^†^
HS + PVN scrambled siRNA	366 ± 10	162 ± 6^∗^	394 ± 13^∗^
NS + PVN PKC siRNA	351 ± 8	103 ± 4	345 ± 7
NE + PVN scrambled siRNA	359 ± 9	97 ± 5	349 ± 8

Values are expressed as means SE. *P < 0.05 *versus* NS groups; ^†^P < 0.05 HS^ ^+ PVN LOS or HS + PVN PKC siRNA *versus* HS control groups.

**Table 2:** Effect of LOS or PKCγ siRNA on MDA, SOD, GSH levels and NAD(P)H oxidase activity in PVN of salt-induced hypertensive and control groups (n = 7, for each group).

PVN	MDA (mmol/mgp)	SOD (U/mgp)	GSH (umol/mgp)	NAD(P)H oxidase (RLU/mg/sec)
HS + PVN LOS	4.87 ± 0.19^†,∗^	6.26 ± 0.30^†,∗^	2.85 ± 0.24^†,∗^	1.91 ± 1.20^†,∗^
HS + PVN aCSF	7.01 ± 0.24^∗^	2.67 ± 0.10^∗^	0.98 ± 0.13^∗^	3.69 ± 1.41^∗^
NS + PVN LOS	1.68 ± 0.22	9.61 ± 0.40	4.76 ± 0.15	0.93 ± 0.34
NE + PVN aCSF	1.25 ± 0.21	10.00 ± 1.40	5.18 ± 0.37	0.87 ± 0.29
HS + PVN PKC siRNA	3.82 ± 0.14^†,∗^	4.57 ± 0.17^†,∗^	1.99 ± 0.18^†,∗^	2.80 ± 1.16^†,∗^
HS + PVN scrambled siRNA	6.28 ± 0.24^∗^	3.67 ± 0.15^∗^	0.98 ± 0.13^∗^	4.27 ± 1.78^∗^
NS + PVN PKC siRNA	2.55 ± 0.13	7.11 ± 0.37	3.24 ± 0.12	1.33 ± 0.32
NE + PVN scrambled siRNA	2.68 ± 0.14	7.85 ± 0.44	2.97 ± 0.11	1.18 ± 0.26

Values are expressed as means SE. *P < 0.05 *versus* NS groups; ^†^P < 0.05 HS + PVN LOS or HS + PVN PKC siRNA *versus* HS control groups.

**Table 3:** Rat primers used for real-time PCR.

Rat genes	Forward (5′–3′)	Reverse (5′–3′)
AT1R	CAAAAGGAGATGGGAGGTCA	TGACAAGCAGTTTGGCTTTG
PKCγ	AGGTGCTGAGAGCGAAGCTCCGC	CTTGCCCCTGTCCTTCCTATCTC
Cu/Zn-SOD	GGTGGGCCAAAGGATGAAGAG	CCACAAGCCAAACGACTTCC
GAPDH	AGACAGCCGCATCTTCTTGT	CTTGCCGTGGGTAGAGTCAT

AT1R, angiotensin II type 1 receptor; PKCγ, protein kinase C γ; Cu/Zn-SOD, copper/zinc superoxide dismutase; GAPDH, Glyceraldehyde 3-phosphate dehydrogenase.
